# Preparation and Characterization of Carrageenase Immobilized onto Polyethyleneimine-Modified Pomelo Peel

**DOI:** 10.4014/jmb.2304.04029

**Published:** 2023-06-19

**Authors:** Qin Yin, Christopher G. Batbatan, Yongxing Li, Yonghui Zhang, Qiuming Yang, Anfeng Xiao

**Affiliations:** 1 College of Biological and Food Engineering, Suzhou University, Suzhou, Anhui, 234000, P.R. China; 2 Department of Biology, Central Mindanao University, Maramag, Bukidnon, 8710, Philippines; 3 College of Ocean Food and Biological Engineering, Jimei University, Xiamen, Fujian, 361021, P.R. China

**Keywords:** Pomelo peel, polyethyleneimine, carrageenase, immobilization

## Abstract

In this study, carrageenase immobilization was evaluated with a concise and efficient strategy. Pomelo peel cellulose (PPC) modified by polyethyleneimine (PEI) using the physical absorption method was used as a carrier to immobilize carrageenase and achieved repeated batch catalysis. In addition, various immobilization and reaction parameters were scrutinized to enhance the immobilization efficiency. Under the optimized conditions, the enzyme activity recovery rate was more than 50% and 4.1 times higher than immobilization with non-modified pomelo peels. The optimum temperature and pH of carrageenase after immobilization by PEI-modified pomelo peel, at 60°C and 7.5 respectively, were in line with the free enzyme. The temperature resistance was reduced, inconsistent with free enzyme, and pH resistance was increased. A significant loss of activity (46.8%) was observed after reusing it thrice under optimal reaction conditions. In terms of stability, the immobilized enzyme conserved 76.0% of the initial enzyme activity after 98 days of storage. Furthermore, a modest decrease in the kinetic constant (*K*_m_) value was observed, indicating the improved substrate affinity of the immobilized enzyme. Therefore, modified pomelo peel is a verified and promising enzyme immobilization system for the synthesis of inorganic solvents.

## Introduction

Carrageenans comprise an important class of linear sulfated polysaccharides that represent the main cellular component of red algae (Rhodophyta)-related seaweeds. They have a repeating main chain of 3-linked β-D-galactose and 4-linked α-D-galactose carrying 3,6-anhydro residues (DA-units) [1]. Hydrocolloid polysaccharides such as carrageenan are pivotal to the food, biotechnological and cosmetic industries due to their exceptional biocompatibility and distinct physicochemical characteristics in emulsifying, thickening, gelling, and stabilizing agents [2]. Moreover, green biotechnology bacterial enzymes are central to hydrolyzing carrageenans into (kappa) κ-, (iota) ι-, and (lamda) λ-carrageenans, which have been developed through modern research to possess pharmacological properties and potential therapeutic applications [3]. Thus far, numerous carrageenases have been isolated and studied, though many aspects of their structure and mechanism remain to be elucidated [4].

Enzyme immobilization is a strategy for limiting or locating enzyme molecules within a certain region or fixed space and maintaining their catalytic activity. It enables enzymes to be used repeatedly over multiple reaction cycles and is the simplest way to solve the problems of rigorous cost controls, solubility in aqueous solution, and instability under reaction conditions in which enzymes take center stage [5]. Various kinds of materials have been utilized as carriers, matrices, or support for immobilizing enzymes. Immobilization requires physical attachment of the enzyme to a water-insoluble matrix/supporting material/carrier, in which case the enzyme is unable to move due to linkage and phase differences between substrates and products [6]. In comparison with free enzymes, immobilized enzymes exhibit enhanced stability and selectivity, easier product recovery and purification, reusability, and continuous process [7].

Recently used and proven methods of enzyme immobilization with various support materials include physical adsorption, covalent bonding, ion exchange, microencapsulation, and matrix entrapment [8]. Among the available support materials, celluloses, by virtue of their high hydrophilicity, biocompatibility, biodegradability, and low risk of environmental contamination, have gained recognition as excellent carriers [9]. Remarkably, this trend has become evident across enzyme immobilization areas, with a reported rise in appropriate immobilization protocols and activated supports by extremely intense covalent attachment [10]. The most widely sourced natural polymer on earth, pomelo (*Citrus grandis* L. Osbeck) peel, principally consists of cellulose, carotenoids, limonoids, flavonoids, essential oils and pectin [11], which makes up the majority of pomelo wastes (50%, w/w)[12]. According to previous studies on the effective yield of pomelo peel, this material could increase the value of cellulose.

In enzyme immobilization, chemical coupling agents should be used to modify the surface of the matrix to endow it with the appropriate functional groups for the sake of improving binding efficiency. A variety of polymers that have amino pendant groups were frequently referred to as suitable enzyme carriers because they usually demonstrate enormous advantages in terms of good water solubility, high functional group content, desirable molecular weight, and promotion of physical and chemical stabilities. PEI has surfaced as a critically studied amine-based cationic polymer, which could give rise to curious situations, as it did in the case of industrial immobilized biosystems [13]. PEI comes in two forms: the linear crystalline type and the more significant amorphous branched structure with a distribution of primary, secondary, and tertiary amino groups at a ratio of 1:2:1 [14]. The primary and secondary amino groups are subjected to modification to generate facile enzyme carriers. The ongoing patents and research have demonstrated how PEI can bind a rich variety of enzymes and whole cells. Velasco‐Lozano *et al*. (2017) introduced the successful “co-immobilization” of enzymes and phosphorylated cofactors through a novel architecture where enzymes and PEI are firmly affixed to the surface of porous materials [15]. PEI needs to be taken more into account, especially when PEI is grafted to the supports and cross-linked by bifunctional agents that are widely used for immobilizing various enzymes, such as lipase [16], β-galactosidase [17], and tyrosinase [18].

In this research we sought to provide a complete and well-tested system for preparing immobilized carrageenase from *Pseudoalteromonas carrageenovora* onto a novel cellulose support functionalized with PEI using the physical absorption method. The enzymatic properties of the free and immobilized enzymes and the factors affecting their activity were analyzed, and the morphological characteristics after immobilization were evaluated.

## Materials and Methods

### Materials

Analytically pure NaH_2_PO_4_•2H_2_O, Na_2_HPO_4_•12H_2_O, citric acid, and sodium citrate were acquired from Sinopharm Chemical Reagent Co., Ltd. Carrageenan was supplied by Green New (Fujian) Food Co., Ltd. Polyethyleneimine was purchased from Xiangbo Biotechnology Co., Ltd., and pomelo peel was purchased from Xiangfa Medicine (China). Sinopharm Chemical Reagent Co., Ltd. provided all other reagents (analytical grade).

### Culture Medium

The appropriate concentration range of the initial medium component was determined as follows: beef extract 10 g, peptone 10 g, distilled water 250 ml, artificial seawater (NaCl 37.51 g, KCl 1.03 g, CaCl_2_ 1.61 g, MgCl_2_·6H_2_O 6.40 g, NaHCO_3_ 0.15 g, MgSO_4_·7H_2_O 4.67 g, distilled water 1,000 ml) 750 ml. After dissolving the beef extract and peptone, the pH was adjusted to 7.8 and the medium was then boiled for 10 min. Then, the medium was adjusted pH 7.3 after cooling, mixed with artificial seawater, and sterilized for 20 min at 121°C.

The fermentation medium consisted of carrageenan 2.5 g, NaCl 30 g, KCl 0.10 g, CaCl_2_ 0.20 g, MgSO_4_·6H_2_O 3.0 g, peptone 3.0 g, FeSO_4_·7H_2_O 0.036 g, NaH_2_PO_4_·2H_2_O 1.3 g, Na_2_HPO_4_·12H_2_O 3.8 g and distilled water 1,000 ml, sterilized for 20 min at 121°C.

### Bacterial Culture and Carrageenase Preparation

*Pseudoalteromonas carrageenovora* ASY5 (GenBank accession no. KJ747189) was the carrageenase-producing strain selected in laboratory. The bacterial cells were cultured in a modified initial medium at 20°C for 24 h before being added to the prepared fermentation medium at a 2% inoculation amount, and fermented at 20°C for 32 h. To separate cells, the liquid culture was centrifuged at 5,000 x *g*, and the collected supernatant, which was the crude enzyme solution, was then stored at 4°C for use.

### Preparation of Immobilized Carrageenase

Conventionally, 0.2 g of well-rinsed pomelo peel is converted into modified support by reacting it with 5 ml of PEI solution (0.2 mg/ml) followed by incubation at room temperature for 2 h. To remove the excess PEI, the carrier was thoroughly washed with phosphate buffer (pH 7.0). Subsequently, the carrier was immobilized at 4°C for 2 h with a mixture of 5 ml phosphate buffer (pH 7.0) and 20 U carrageenase. Finally, to leave pure immobilized carrageenase, the resulting samples were thoroughly washed again with phosphate buffer (pH 7.0) and stored at 4°C to prepare for determining the activity.

### Assay of Enzyme Activity of Free and Immobilized Carrageenase

There were three recommended steps, called the 3,5-dinitrosalicylic acid (DNS) method, for measuring the enzyme activity of free and immobilized carrageenase [19]. The steps were as follows: mix carrageenase with 0.5%(w/v) carrageenan solution, prepared with sodium phosphate buffer (50 mM, pH 7.0), and incubated at 60°C for 20 min. Then, incubate the mixture with boiling water for 5 min after adding DNS into the reaction solution, and measure the absorbance at 520 nm. One unit of carrageenase activity was defined as the amount of enzyme that produced 1 μmol/min of D-galactose under specific conditions (60°C and pH 7.0) where sugar content could be expressed in terms of the D-galactose in the standard curve.

Following immobilization, the residual enzyme activity was enzyme recovery rate. The enzyme activity recovery rate might be tackled according to the following equation:

Recovery rate (%) = activity of the immobilized carrageenase / total activity of the free carrageenase × 100%. Relative activity (%) = activity of each group / the highest activity value in this group × 100%.

### Improvement of Conditions for Carrageenase Immobilization

The process of immobilization needs to be optimized, enabling it to reach maximum potential in the four factors of PEI concentration (0-0.4 mg/ml), adsorption time (0.5-4 h), pH (6.0-8.0), enzyme volume (5-30 U/ml carrageenase), immobilization time (2-10 h) as well as immobilization temperature (4 and 25°C). Experiments were all performed in triplicate while a high recovery rate and relative activity of carrageenase indicated high immobilization efficiency and vice versa.

### Fourier-Transform Infrared Spectra (FTIR) and Scanning Electron Microscopy of Free and Immobilized Carrageenase

To obtain FTIR spectra in the 4,000-400 cm^-1^ region, we turned to a NEXUS 670 FTIR instrument using KBr discs at room temperature (25°C). The morphology of the immobilized carrageenase was revealed by SEM (Hitachi S-4800 Field, Japan).

### Enzymatic Characterization of Free and Immobilized Carrageenase

We conducted a close study of the effect of different temperatures (45°C to 65°C) and pH values and recorded the results from pH 6.0 to 8.0 in the following buffers: Na_2_HPO_4_-NaH_2_PO_4_ (50 mM, pH 6.0 - 7.0) and Tris-HCl (50 mM, pH 7.0-8.0) with an incubation time of 5 min. Not only is the maximum activity deemed to be 100%, but it is also regarded as the standard of relative activity under various experimental conditions. To check the thermal stability bottlenecks of free and immobilized carrageenase, the residual activity of both was measured following storage from 35°C to 55°C for 1 h. The pH stability of both free and immobilized carrageenase was determined by analyzing the residual activities after incubation at 4°C for 24 h in the respective pH buffers. We divided the residual enzyme activity by the initial enzyme activity to calculate the specific enzyme activity of 100%.

### Operation Stability and Storage Stability Analysis

During the reusability program, carrageenan was hydrolyzed by immobilized carrageenase at 45°C and pH 7.5. The free and immobilized carrageenase activity was evaluated by storing at 4°C for 14 weeks to represent storage stability. It is assumed that the relative activity was activity received in each round/initial activity (defined as 100%).

### Kinetic Parameters

The Lineweaver-Burk plot consists of plotted values of 1/V vs. 1/[S], which form the apparent kinetic parameters (*K*_m_) and maximum reaction velocity (*V*_max_) of free and immobilized carrageenase determined using varying κ-carrageenan concentrations from 0.2% to 0.7% (w/v) carrageenan at 60°C and pH 7.5.

### Statistical Analysis

All data were presented as the means ± standard error of the mean (SEM). One-way ANOVA and Duncan’s multiple range test were used to determine statistical significance. A *p*-value of 0.05 or less (*p* < 0.05) indicated statistical significance.

## Results and Discussion

### Determination of Carrageenase Immobilization Methods

The conditions for modifying pomelo peel with potassium periodate, PEI, or both at the same time were as follows: treatment with 5 ml, 2 mg/ml potassium periodate solution for 4 h followed by washing 5 times; then, treatment with 5 ml, 0.2 mg/ml PEI solution for 2 h followed by washing 5 times. Investigations were conducted into the effects of various treatments on enzyme activity and recovery. The results were shown in Table 1.

According to Table 1, when only the PPC was used for fixation, the weak adsorption of the carrier onto the enzyme led to a decrease in enzyme activity. After oxidation with potassium periodate, the hydroxyl group on the fiber is oxidized to an aldehyde group in the enzyme, which bonds covalently with the amino group. Potassium periodate has an oxidizing effect, and therefore pomelo peel oxidized by potassium periodate was expected to better immobilize carrageenase. However, after significance analysis, no significant effect on immobilization with or without potassium periodate oxidation was found. This was due to pomelo peel fiber itself having a large number of aldehyde groups that can be used to immobilize carrageenase molecules and facilitate the immobilization of the carrageenase [20]. Girelli and Scuto (2021) found that the immobilized laccase activity leaked 82% after treatment with potassium periodate comparing other immersion method procedures [21].

When treatment with potassium periodate is not used for oxidation, the enzyme activity is low, and the enzyme activity can be effectively improved through PEI treatment. The low enzyme activity may be caused by the low number of aldehyde groups or the inability of the aldehyde group to effectively bind to the enzyme protein. Cross-linking hydroxyl groups in PEI to fiber coupling, high cationic characteristics, formation of strong adsorption of enzyme protein, and the carrier surface grafting polyethylene imine can provide greater surface area for adsorption of the enzyme, resulting in the immobilized enzyme exerting relatively high activity. Therefore, in the subsequent experiments only polyethyleneimine was used to deal with pomelo peel. The immobilization enzyme activity and recovery rates were 24.0 U/g and 24.0%, respectively.

Among natural polymers, cellulose, chitosan, alginate, and their derivatives are often utilized in enzyme immobilization. Cellulose is one of the best carriers for enzyme immobilization. These materials possess distinguishing features like extremely fine network architecture with a distinct tunnel, porous structure, significant specific surface area, and exceptional mechanical strength.

Cai *et al*. prepared immobilized lipases using aldehyde-modified, sphere-like bacterial cellulose as a green carrier for better hydrolytic oil recycling across many different industries [22]. *Pseudomonas cepacian* lipase was immobilized onto magnetic cellulose nanocrystals to increase the enzyme structure rigidity compared with free enzyme [23]. Bovine pancreas trypsin could be immobilized most effectively by cross-linking to magnetic bacterial cellulose that had been activated with sodium periodate. This biocatalyst was very stable and maintained its enzyme activity for 10 cycles [24]. As a carrier for laccase, rotating magnetic field-exposed bacterial cellulose could perform effectively [25]. Physical absorption immobilizes enzymes [26]. Caterina G.C *et al*. (2018) immobilized FalDH and ADH enzymes on magnetic nanoparticles via the same adsorption mechanism applied to perform the CO_2_/methanol conversion cycle [27]. Novel nano-bioconjugates, different from conventional immobilized enzymes, were conferred broader applications or could be used for continuous catalytic processes due to their distinct physicochemical properties [28]. Different methods such as surface adsorption, covalent binding, diffusion, and co-precipitation can be used to immobilize different enzymes and metal-organic frameworks [29]. At the molecular level, the interaction between enzymes and carriers is like the relationship between proteins and supporting surfaces. From short peptides to large proteins, different immobilization techniques can interrogate protein orientation and conformation [30].

### FTIR Spectroscopy and Morphology of Carrageenase and Immobilized Carrageenase

**Fig. 1:**
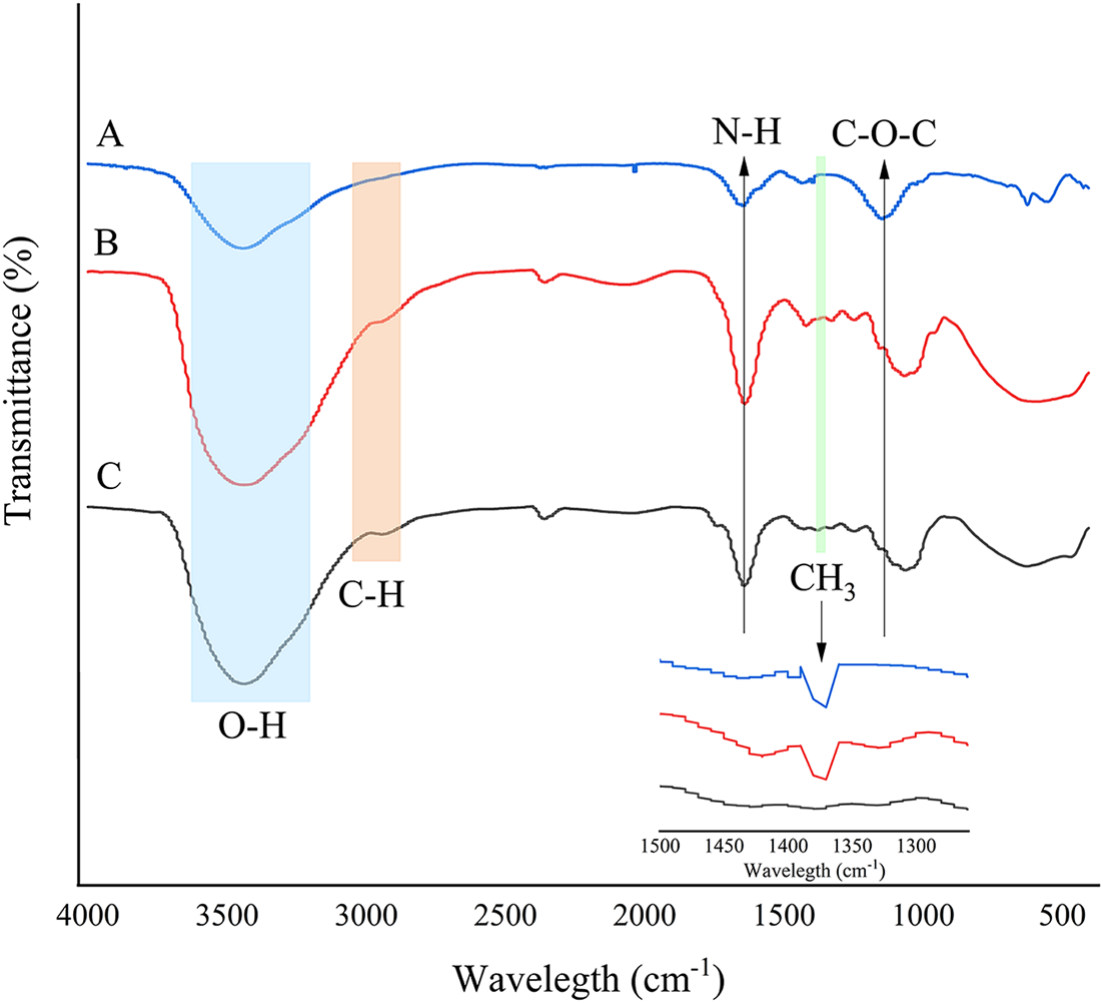
FTIR spectra of free carrageenase (A), immobilized carrageenase on PPC (B) and PPC (C).

FTIR was used to determine the structures of free carrageenase (A), immobilized carrageenase on PPC (B), and PPC (C) (Fig. 1). The vibrational peaks around 3,650 to 3,200 cm^-1^ on the pomelo peel carrier and immobilized carrageenase were attributed to the O-H. In addition, there were characteristic absorption bands around 3,650 to 580 cm^-1^ for free O-H groups, which are a strong polar group easily associated. The hydroxyl group is a strong polar group and is prone to association, forming a wider absorption band. The stretching vibration absorption band of C-H at 3,100 to 3,000 cm^-1^ overlapped with this absorption band, thus forming a wider absorption band at 3,650 to 3,000 cm^-1^. Absorption peaks at 1,567 cm^-1^ and 1,020.1 cm^-1^ reflect the deformation vibration of N-H groups and the stretching vibration of C-O-C, respectively. The above absorption peaks appeared in all three spectra, indicating that modified PPC, immobilized enzymes and free enzymes all have the same stretching and vibrating groups. The immobilized and free carrageenase both exhibited a peak around 1,370–1,380 cm^-1^, which was caused by the symmetric deformation vibration of CH_3_. To the contrary, PPC lacks this peak. In accordance with the findings, carrageenase was effectively fixed on modified PPC.

### Effects of Immobilization Parameters

**Fig. 2:**
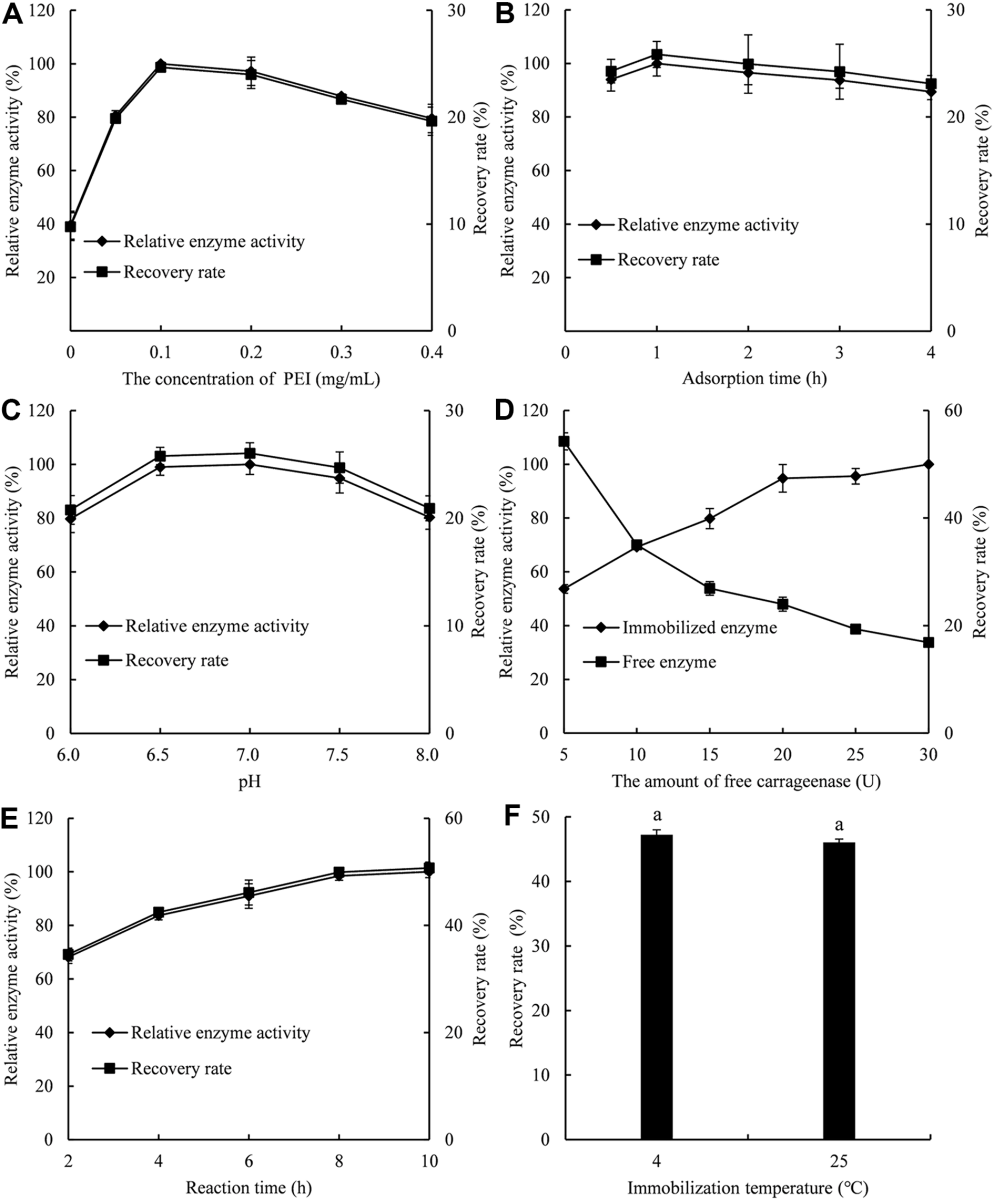
Effect of various process conditions on the immobilization of carrageenase. Different letter indicates significant difference at *p* < 0.05 using Duncan’s multiple range test.

The final yield of immobilized carrageenase depends heavily on the optimal physical absorption conditions, including the amount of PEI and catalyst as well as the reaction pH, time, and temperature. As Fig. 2A shows, 0.2 g of pomelo peel was activated using free carrageenase (10 U) as the catalyst at different concentrations of PEI. The maximum immobilized efficiency of carrageenase was obtained at 0.1 mg/ml. Experiments were repeated to confirm that 0.1 mg/ml PEI provides the most stable working condition for pomelo peel. Therefore, in subsequent studies, PEI-assisted cellulose immobilization was used at a concentration of 0.1 mg/ml.

As depicted in Fig. 2B, adsorption time with PEI has a great influence on the immobilization efficiency. We observed that carrageenase from *P. carrageenovora* ASY5 was rapidly fixed to the modified PPC. A sizable amount of extremely active enzymes (85%) could be immobilized within the first 30 min of the adsorption reaction. Carrier modification occurred instantly. However, after an obvious activity peak in the first 60 min of adsorption, the enzyme activity essentially remained stable after that. It is evident from Fig. 2B that, following a 2 h adsorption period, the enzyme activity slightly dropped as a result of diffusion limitation brought on by bulk immobilization, which prevented any additional enzymes from making contact with the carriers. Therefore, the highest carrageenase activity was found after an adsorption time of 1 h, indicating faster reaction times and more efficient enzyme immobilization.

The pH value made a great difference to the immobilized enzyme activity. In the pH range of 6.0–7.0, the amount of adsorption with enzyme activity above 80% was clearly visible in Fig. 2C. However, with the increase of pH value, the enzyme partly lost its activity, resulting in the loss of immobilization efficiency. At pH 7.0, immobilization was most efficient. Immobilized pH values had more influence on enzyme activity in an acidic environment than in an alkaline environment. At acidic to neutral pH, the cationic density of PEI is higher and there was a negative potential that appears in the active site in carrageenase resulting in higher activity towards enzyme immobilization and hence, the vice versa activity at alkaline pH, resulting in reverse reaction, can be expected.

From the perspective of sustainable development, optimizing the amount of free carrageenase makes it possible to use the least amount of catalyst while maximizing the yield of immobilized carrageenase. Carrageenase was immobilized by varying amounts of free carrageenase, ranging from 5 to 30. Fig. 2D shows that the optimal amount of free carrageenase was 10 U. Under the existing conditions, a higher amount had no effect on the recovery rate of immobilized carrageenase. In contrast, an abundance of enzymes could lead to overloading of the support, leading to a decrease in enzyme activity [31].

Following the determination of the optimal reaction temperature and amount of free carrageenase, the optimal reaction time was found. At 50°C, 0.2 g of pomelo peel was activated with free carrageenase (10 U) for miscellaneous reaction times (2–10 h). Carrageenase immobilized for 8 h was most effective. Pomelo peel had to be modified for at least 8 h to achieve 97% recovery rate, while 80% recovery rate could be obtained in 4 h (Fig. 2E). For consistency, 8 h was the best reaction time in this study.

For the stability and catalytic activity of enzymes, the immobilization temperature is critical. Fig. 2F shows how temperature affects enzyme immobilization and activity. The immobilization process at 25°C was no different from 4°C and their recovery rate was 46.4% and 47.8%, respectively. Carrageenases are immobilized at 25°C to reduce costs and conserve resources.

Cao *et al*. (2020) investigated the effect of reaction time, temperature, and pH on PEI-cross-linked lipase [32]. Bezerra *et al*. (2020) presented a study of the immobilization conditions including pH and the incubation time [33].

### Enzymatic Properties of Free and Immobilized Carrageenase

**Fig. 3:**
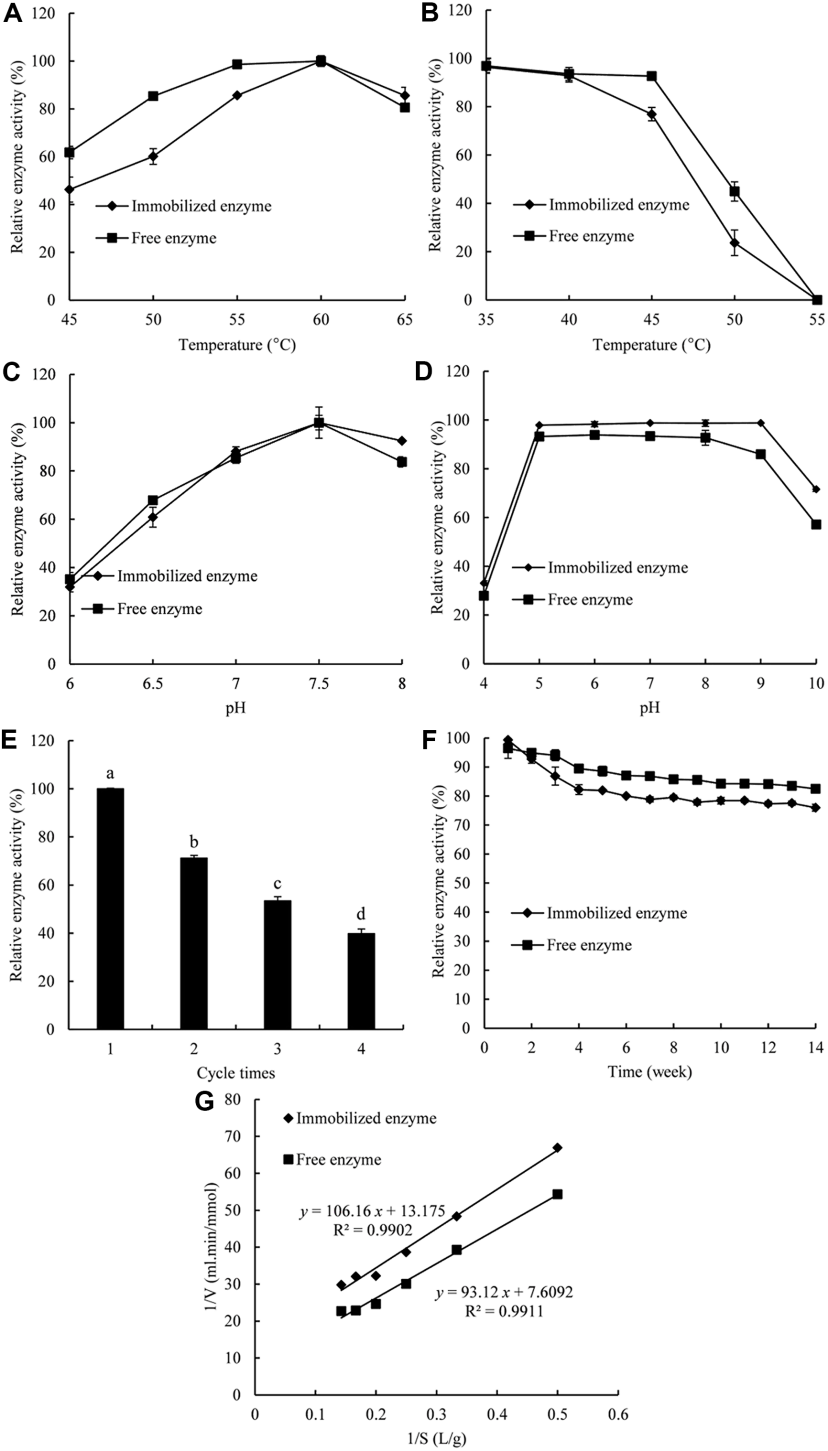
Optimum reaction temperature (A) temperature stability (B) optimal pH (C) pH stability (D) reusability (E), storage stability (F) and Lineweaver-Burk plot (G) of both free and immobilized enzyme. Different letter indicates significant difference at *p* < 0.05 using Duncan’s multiple range test.

By carrying out catalytic reactions ranging from 45°C to 65°C, we were able to determine the temperature at which free and immobilized carrageenases had the best chance of achieving their optimal reaction. Due to the changes in physicochemical properties caused by immobilization, as depicted in Fig. 3A, the optimal temperature of immobilized carrageenase was lower than that of the free enzyme. After covalent binding of the enzyme to the supporting matrix, the diffusion restriction was increased, and the activity of carrageenase was not further improved after immobilization [34]. At relatively high temperatures ranging from 55 to 60°C, the immobilized enzyme’s activity remained above 80%. However, the activity of the enzyme suddenly decreased since the reaction temperature was not 60°C. In terms of stability, free enzymes outperform immobilized enzymes in the same temperature range. The conformational mobility of the molecule after immobilization of the enzyme with the substrate was limited so that the stability of immobilized carrageenase was lower than that of free carrageenase [35].

The thermal stability of both free and immobilized carrageenase was determined at low temperature range (35–55°C). Free carrageenase retained over 99.2% initial activity after 1 h incubation at 35–45°C, while the immobilized counterpart retained only about 80%, as illustrated in Fig. 3B. The residual activity of free carrageenase was about 43.8% when the incubation temperature was increased to 50°C, compared to only 23.3%for immobilized carrageenase. The immobilized form of carrageenase was not as stable as the free form, likely because of the reduced conformational mobility of the molecules [36].

The catalytic activity of both free and immobilized enzymes was compared as the pH varied. The pH-activity profiles of the free and immobilized enzyme are depicted in Fig. 3C. At an approximate pH of 6.0, the enzyme activity reached the bottom. When the pH value rose to 7.0–8.0, the activity of immobilized carragenase also increased, reaching more than 80%. The optimum pH of the immobilized enzyme was not altered compared to the free enzyme with an alkaline value of 7.5. The tolerance of the immobilized enzyme to acids and alkalis was consistent with free enzyme because the microenvironment around the enzyme molecule is changed, and the pH of the microenvironment around the charged groups of the polyelectrolyte PEI is different from that of the overall solution. The high hydrophilicity of PEI also helps to protect the hydrated layer on the enzyme surface and avoid enzyme inactivation. After immobilization, the binding of PEI to the enzyme protein reduces the entry of external ions and maintains the pH of the microenvironment with relative stability. Immobilization of *Thalassospira* sp. fjfst-332 κ-carrageenase onto a magnetic Fe_3_O_4_-chitosan carrier did not result in an optimal pH change [37]. On the other hand, the results from Mohammadi *et al*. [38] and Ates *et al*. [39] indicated that the optimal pH was shifted to an acidic value after immobilization.

Under different pH values (4.0–10.0), immobilized carrageenase had significantly greater pH stability than free carrageenase (Fig. 3D). This finding can be attributed to the microenvironmental structure and specific enzyme protection properties formed by pomelo peel as a carrier. Immobilized enzymes are less affected by pH than free enzymes because of the cushioning effect of the microenvironment. Notably, free and immobilized carrageenase were more productive at nearly neutral pH and began to lose activity in acidic or alkaline environments. As already pointed out in previous reports, enzyme immobilization is aimed at improving the stability of the enzyme [22]. The immobilized carrageenase maintained more than 92.4% of its initial activity. In this respect, it had much higher pH stability. Unfortunately, the residual activity of free carrageenase differed decreasing to around 83.7%. Since the conformation of carrageenase immobilized on the novel PPC was more stable, there was broader pH tolerance.

### Reusability and Storage Stability

Immobilized enzymes are credited with playing a central role in saving production costs through repeated, continuous, and batch uses. Kaur *et al*. (2021) announced that cellulase immobilized onto magnetic nanoparticles for enzymatic saccharification of rice straw exhibits 50.34% residual activity after four reactions [40]. This finding is consistent with our study, which showed 53.4% of residual activities for κ-carrageenase immobilized on magnetic nanoparticles after three cycles, and activity that gradually bogged down in successive cycles (Fig. 3E).

A series of changes in storage stability of free and immobilized carrageenase has been pushed forward since biocatalysts were stored at 4°C and the stability was determined at 7-day intervals. As shown in Fig. 3F, free carrageenase showed better retention of its initial activity following immobilization. After 98 days of storage, the free carrageenase preserved 82.5% of its initial activity, while the corresponding value for immobilized carrageenase was 76.0%. The immobilized carrageenase contended with an enzyme activity downturn resulting from modification of the 3D structure of the enzyme after immobilization. In comparison, Nimkande and Sivanesan (2023) reported that *Bacillus altitudinis* Ant19 lipase showed 38.9% of original activity and immobilized lipase preserved 51.1% of its original activity [41].

### Kinetic Parameters

We examined the reactivity of free and immobilized enzymes, primarily focusing on the kinetic parameters *K*_m_ and *V*_m_. The Michaelis-Menten constants *K*_m_ and *V*_m_ for the free enzyme have been determined to be 11.98 g/l and 0.131 mmol/ml/min, respectively (Fig. 3G). *K*_m_ was decreased to 8.07 g/l mM and *V*_m_ plunged to 0.076 mmol/ml/min after the enzyme was immobilized onto modified pomelo peel. In the immobilized system, the enzyme molecule and carrier were confined with chemical modification, which is advantageous for preserving the inherent affinity to substrate molecules. Underneath that, for the same amount of soluble enzyme, a lower concentration of substrate is required to improve interactions with the immobilized enzyme. This has caused a decrease in *K*_m_. The formation of linkages between carrageenase and support restricted the mobility of the enzyme, thereby suppressing its unfolding due to environmental pressures, resulting in a reduction of the maximum reaction velocity *V*_m_. Asad *et al*. [42] designed water-insoluble ethylcellulose-based fibers containing encapsulated α-amylase. The *K*_m_ value of immobilized enzyme (4.237 mg/ml) was lower than that of free enzyme (5.56 mg/ml), indicating that the affinity of α-amylase increased after immobilization into the fiber. The *V*_m_ values for free enzyme and immobilized enzyme were 2.14 and 1.90 μmol/ml/min, respectively.

## Conclusion

Bifunctional enzyme carrageenase was immobilized onto PPC. The immobilized carrageenase possesses improved thermal and storage stability, with excellent stability during storage, retaining more than 82.5% activity over 98 days. In terms of pH, the enzymatic characteristics of the immobilized carrageenase fusions were comparable to those of free enzymes, exhibiting the same optimal pH. There was only a modest decrease in *K*_m_, meaning immobilized enzymes have better substrate affinity. Overall, our study establishes the applicability of crude and immobilized carrageenase preparation, which should be scaled up further.

## Figures and Tables

**Table 1:** Treatment-related methods on the immobilization of carrageenase.

Potassium periodate oxidized or not	Treated with PEI or not	Enzyme activity (U/g)	Enzyme activity recovery rate (%)
No	No	9.1 ± 0.3^b^	9.1 ± 0.3^b^
Yes	Yes	22.7 ± 0.7^a^	22.7 ± 0.7^a^
No	Yes	24 ± 0.9^a^	24 ± 0.9^a^
Yes	No	8.8 ± 0.3^b^	8.8 ± 0.3^b^

The data are presented as the means ± SEM. Significant differences (*p* < 0.05) between groups as determined by Duncan’s multiple range test are indicated with different letters.
